# Atherosclerotic cardiovascular disease risk is associated with AT(N)V neuroimaging biomarkers and cognitive impairment in the health and aging brain study–health disparities cohort

**DOI:** 10.3389/fnagi.2026.1874472

**Published:** 2026-10-05

**Authors:** Kimberly Espejo, Ryan Rulkens, Cellas A. Hayes, Joey A. Contreras

**Affiliations:** 1 Department of Neurosciences, Alzheimer’s Disease Cooperative Study (ADCS), University of California San Diego, San Diego, CA, United States; 2 Department of Biological Sciences, University of California San Diego, San Diego, CA, United States; 3 Halıcıoğlu Data Science Institute, University of California San Diego, San Diego, CA, United States; 4 Sanders-Brown Center on Aging, College of Medicine, University of Kentucky, Lexington, KY, United States; 5 Department of Neuroscience, College of Medicine, University of Kentucky, Lexington, KY, United States

**Keywords:** aging, Alzheimer’s disease, AT(N)V framework, atherosclerotic cardiovascular disease, cognitive impairment, neuroimaging biomarkers, white matter hyperintensities

## Abstract

**Background:**

Cardiovascular risk is increasingly recognized as relevant to cognitive aging and dementia-related brain changes, yet its relationship to multimodal neuroimaging biomarkers of amyloid, tau, neurodegeneration, and vascular injury (AT(N)-V) remains incompletely understood in racially and ethnically diverse cohorts. We examined whether ten-year atherosclerotic cardiovascular disease (ASCVD) risk was associated with AT(N)-V neuroimaging biomarkers and cognitive domain scores.

**Methods:**

This cross-sectional study included 1,626 Health and Aging Brain Study–Health Disparities (HABS-HD) participants without cardiovascular disease or dementia who had amyloid positron emission tomography (PET) imaging linked to visit 1. Ten-year ASCVD risk was estimated using the Pooled Cohort Equations and modeled continuously. Neuroimaging outcomes included global florbetaben amyloid PET standardized uptake value ratio (SUVR), medial temporal lobe (MTL) PI-2620 tau PET SUVR, cortical thickness, hippocampal volume, and white matter hyperintensity (WMH) volume. Cognitive domain outcomes included memory, executive function, processing speed, and language. Multivariable linear regression models accounted for apolipoprotein E (*APOE*) ε4 status, relevant neuroimaging biomarkers, and imaging scanner/protocol covariates as appropriate, with Holm adjustment for multiple comparisons. Amyloid-status-stratified analyses were also conducted using a global amyloid PET SUVR threshold of 1.08.

**Results:**

Increasing ten-year ASCVD risk was associated with increasing global amyloid and MTL tau PET SUVR, smaller cortical thickness, smaller hippocampal volume, and increased WMH volume. Increasing ten-year ASCVD risk was also associated with lower memory, executive function, processing speed, and language scores after accounting for APOE ε4 status, amyloid burden, neurodegeneration, vascular injury, and scanner covariates. Associations between ten-year ASCVD risk and structural and vascular injury markers were observed in both amyloid-positive and amyloid-negative participants, whereas associations with amyloid and MTL tau PET were modest and statistically significant only among amyloid-negative participants.

**Conclusion:**

In this diverse HABS-HD sample, increasing ten-year ASCVD risk was associated with greater AT(N)-V biomarker burden and poorer cognitive performance across multiple domains. These cross-sectional findings suggest that ten-year ASCVD risk may serve as a marker of co-occurring cardiovascular and brain health burden, but they do not establish causality or temporal direction.

## 1 Introduction

Alzheimer’s disease (AD) and related dementias are increasingly recognized as multifactorial conditions influenced by both neurodegenerative pathologies and vascular processes (Schneider et al., 2007; Montine et al., 2012). While traditional models of AD have emphasized amyloid and tau pathology as central drivers of cognitive decline, there is growing evidence that vascular risk factors also play a critical and potentially independent role in cognitive decline and dementia risk (Iadecola, 2013). Extensive research has shown that cardiovascular conditions, including hypertension, diabetes, and dyslipidemia, are consistently associated with poorer cognitive outcomes and increased risk of incident dementia (Crane et al., 2013; Gottesman et al., 2017a; Livingston et al., 2020; Hayes et al., 2025a), highlighting the importance of vascular health in brain aging.

In parallel with this shift, the AT(N) framework has provided a biologically grounded approach for characterizing AD through biomarkers of amyloid deposition (A), tau pathology (T), and neurodegeneration (N) (Jack et al., 2018). More recent work has suggested that additional processes, including vascular injury (V) and inflammation (I), may also contribute meaningfully to disease expression and heterogeneity. In the present study, however, we focus on the AT(N)V framework, which incorporates vascular injury to better capture the contribution of cerebrovascular disease to cognitive impairment (Nation et al., 2019). This framework reflects the recognition that vascular and neurodegenerative processes often co-occur and jointly contribute to disease progression (Beach et al., 2012; Jack et al., 2024; Hayes and Younes, 2025). However, despite this conceptual integration, the extent to which global cardiovascular risk influences each component of the AT(N)V cascade remains incompletely understood.

An estimated ten-year atherosclerotic cardiovascular disease (ASCVD) risk score, calculated using the Pooled Cohort Equations, provides an integrated estimate of cardiovascular risk based on demographic and cardiometabolic factors (Goff et al., 2014). Prior work has shown that individual vascular risk factors, such as hypertension and diabetes, are associated with structural brain changes, white matter injury, and cognitive decline (Debette et al., 2011). Emerging evidence further suggests that vascular risk may be associated with amyloid and tau pathology, although findings have been mixed and are often limited by smaller samples or the absence of multimodal biomarker data (Rodrigue et al., 2013; Pillai et al., 2016). As a result, few studies have examined whether a global and predictive measure of cardiovascular risk relates to the full AT(N)V framework within the same cohort.

Higher estimated 10-year ASCVD risk has been associated with cognitive impairment independent of traditional AD markers, suggesting that cardiovascular risk burden may be related to cognitive outcomes through pathways not fully captured by canonical AD pathology (Pettigrew et al., 2020). However, it remains unclear whether the relationship between ten-year ASCVD risk and cognition reflects its association with AT(N)V biomarkers or whether ten-year ASCVD risk contributes additional information beyond these neuroimaging measures. Clarifying this distinction is important for understanding whether vascular risk acts primarily through downstream brain injury, through overlap with AD-related processes, or through both pathways.

These questions are particularly important in racially and ethnically diverse populations, where both cardiovascular disease burden and dementia risk are disproportionately elevated (Martin et al., 2025; Writing Committee Members et al., 2025; Alzheimers Dement, 2026). This is particularly relevant to the present analysis because race and ethnicity are incorporated directly into the Pooled Cohort Equations used to estimate ten-year ASCVD risk. At the same time, most biomarker discovery and validation studies have been conducted in predominantly non-Hispanic White cohorts, limiting generalizability and potentially obscuring important sources of heterogeneity in disease mechanisms (Mayeda et al., 2016). Examining ten-year ASCVD risk within a diverse cohort may therefore provide a more clinically relevant understanding of how vascular burden relates to AD-related and non-AD-related brain changes.

The present study addresses these gaps by examining the relationship between ten-year ASCVD risk and AT(N)V neuroimaging biomarkers in a large, diverse cohort, the Health and Aging Brain Study–Health Disparities (HABS-HD). In this cross-sectional study, we evaluated (1) whether the ten-year ASCVD risk is associated with AT(N)V neuroimaging biomarkers; (2) whether ASCVD risk is associated with domain-specific cognitive performance after accounting for AT(N)V biomarkers; and (3) whether these associations differed by amyloid positivity status. We hypothesized that elevated ten-year ASCVD risk would be associated with greater AT(N)V biomarker burden and poorer cognitive performance across domains, particularly executive function and processing speed, with amyloid positive-stratified analyses providing additional context for interpreting tau-related findings.

## 2 Methods

### 2.1 Study population

Data were obtained from the Health and Aging Brain Study–Health Disparities (HABS-HD), an ongoing longitudinal cohort designed to examine contributors to AD and related dementias in a racially and ethnically diverse population (Petersen et al., 2025). HABS-HD enrolls adults aged 30 and older who are fluent in English or Spanish and self-identify as non-Hispanic White (NHW), non-Hispanic Black (NHB), or Hispanic (O’Bryant et al., 2021; Petersen et al., 2025). Participants undergo harmonized clinical, neuropsychological, neuroimaging, and biomarker assessments. The HABS-HD protocol begins with a clinical baseline visit (visit 1) followed by longitudinal follow-up visits at 24 months intervals. Each study visit includes multiple standardized evaluations which are intended to be completed within 3 months, with a completion window of up to 6 months. These evaluations include demographic and health questionnaires, comprehensive cognitive testing, blood collection, structural magnetic resonance imaging (MRI), and positron emission tomography (PET) imaging for amyloid and tau. Cognitive diagnoses are determined through a multidisciplinary consensus adjudication process (Petersen et al., 2025). All participants provided written informed consent, and study procedures were approved by the University of North Texas Health Science Center Institutional Review Board in accordance with the Declaration of Helsinki (Petersen et al., 2025).

#### 2.1.1 Inclusion criteria for analytical sample

The received dataset included 4,134 participants who completed visit 1, defined as the baseline HABS-HD clinical assessment (Petersen et al., 2025). To align cardiovascular risk estimation with neuroimaging and cognitive measures obtained as part of the baseline study visit, participants were required to have an amyloid PET scan with an available global cortical florbetaben standardized uptake value ratio (SUVR) measure linked to visit 1. Imaging was linked according to the HABS-HD visit structure rather than using a separate *post hoc* fixed time interval between the clinical assessment and PET scan. The visit 1 clinical assessment served as the anchor visit, MRI data were linked to the corresponding clinical visit, and amyloid PET data were linked to the corresponding MRI used for PET SUVR registration. Under the HABS-HD protocol, assessments comprising a study visit are intended to be completed within 3 months, with an allowable completion window of up to 6 months (Petersen et al., 2025). Amyloid burden was quantified using this global cortical summary measure.

A total of 2,165 participants did not meet this imaging criterion and were excluded, leaving 1,969 participants. Some excluded participants had amyloid PET imaging linked to a later study visit rather than to visit 1, particularly individuals originally enrolled through the Health and Aging Brain among Latino Elders (HABLE) study before the study protocol expanded into HABS-HD. Participants with a history of clinical cardiovascular disease were excluded because the pooled cohort equations are designed to estimate ten-year ASCVD risk in a primary-prevention population without established cardiovascular disease. This exclusion removed 126 participants, leaving 1,843 participants. An additional 102 participants with a clinical diagnosis of dementia were excluded, leaving 1,741 participants. We then excluded 24 participants with missing systolic blood pressure measurements and 91 participants with missing data for one or more other components required to calculate the Pooled Cohort Equation for calculating 10-year ASCVD risk score. The final analytic sample included 1,626 participants. Missing data for individual neuroimaging and cognitive outcomes among these participants are reported in Table 1, and the available sample size is provided for each statistical model.

**Table 1:** HABS-HD analytical sample at clinical visit 1.

Variable	Value
ASCVD Risk Score, Mean (SD)	0.15 (0.13)
ASCVD Risk Score, Median [Min, Max]	0.11 [0, 0.90]
Age (years), Mean (SD)	63.5 (8.16)
Male, *n* (%)	569 (35.0)
Race and Ethnicity, *n* (%)	
Non-Hispanic Black	810 (49.8)
Hispanic	424 (26.1)
Non-Hispanic White	392 (24.1)
Diabetes, *n* (%)	401 (24.7)
Hypertension, *n* (%)	1,095 (67.3)
Tobacco Dependence, *n* (%)	136 (8.4)
Total Cholesterol (mmol/L), Mean (SD)	4.69 (1.08)
Total HDL (mmol/L), Mean (SD)	1.48 (0.40)
APOE ε4 Status, *n* (%)	519 (31.9)
Missing, *n* (%)	87 (5.4)
Global Amyloid PET SUVR, Mean (SD)	1.03 (0.13)
Global Amyloid PET SUVR Positivity (> = 1.08), *n* (%)	247 (15.2)
Amyloid PET Scanner, *n* (%)	
Vision	1,597 (98.2)
MCT20	28 (1.7)
Missing, *n* (%)	1 (0.1)
MTL Tau SUVR Mean (SD)	1.13 (0.12)
Missing, *n* (%)	749 (46.1)
Tau PET Scanner, *n* (%)	
Vision	897 (55.2)
Missing, *n* (%)	729 (44.8)
Cortical Thickness Mean (SD)	2.78 (0.12)
Missing, *n* (%)	108 (6.6)
Intracranial Volume (mL), Mean (SD)	1,420 (140)
Missing, *n* (%)	16 (1.0)
Hippocampal Volume (mL), Mean (SD)	6.45 (0.75)
Missing, *n* (%)	168 (10.3)
Hippocampal Volume, ICV-adjusted *z*-score Mean (SD)	0.07 (0.94)
Missing, *n* (%)	168 (10.3)
Raw WMH Volume (mL), Mean (SD)	2.77 (5.58)
Missing, *n* (%)	96 (5.9)
Log WMH Volume (ICV-adjusted), Mean (SD)	−7.38 (1.60)
Missing, *n* (%)	110 (6.8)
MRI Scanner, *n* (%)	
Skyra	31 (1.9)
Vida1	1,536 (94.5)
Vida1a	28 (1.7)
Missing, *n* (%)	31 (1.9)
Mild cognitive impairment, *n* (%)	435 (26.8)

Participant characteristics are presented for the analytic sample (*N* = 1,626). Continuous variables are reported as mean ± standard deviation (SD), and categorical variables are presented as counts and percentages. ASCVD risk was estimated using the American College of Cardiology/American Heart Association Pooled Cohort Equations and categorized for descriptive purposes only. Neuroimaging biomarkers include global amyloid positron emission tomography (PET) standardized uptake value ratio (SUVR), medial temporal lobe (MTL) tau SUVR, cortical thickness (meta-region of interest), hippocampal volume (raw and intracranial volume [ICV]-adjusted *z*-score), and white matter hyperintensity (WMH) volume (raw and log-transformed, ICV-adjusted). Missing values are reported where applicable.

### 2.2 Demographic, clinical, and genetic variables

Age was recorded in years and modeled as a continuous variable. Sex was categorized as male or female, and educational attainment was defined as total years of formal schooling. Race and ethnicity were self-reported and categorized as NHW, NHB, or Hispanic. Cardiometabolic risk factors, including hypertension, diabetes, and smoking status, were defined using self-report and medication use in accordance with established HABS-HD protocols (Hayes et al., 2025a). Genomic DNA was extracted from whole blood samples, and apolipoprotein (*APOE*) genotyping was performed using TaqMan assays targeting rs429358 and rs7412. Participants were classified as *APOE* ε4 carriers, defined as having at least one ε4 allele, or non-carriers.

Resting systolic blood pressure was measured in the seated position using two readings obtained during the same study visit and averaged. Total cholesterol and high-density lipoprotein (HDL) cholesterol were quantified from fasting blood samples and expressed in milligrams per deciliter (mg/dL). Additional cardiometabolic risk factors included dyslipidemia, obesity, and tobacco use, each operationalized as binary variables using previously described HABS-HD definitions (Hayes et al., 2025a). In brief, hypertension and diabetes were defined by self-reported physician diagnosis or use of corresponding medications, dyslipidemia by clinical history or lipid-lowering therapy, obesity by body mass index (BMI) ≥ 30 kg/m^2^, and tobacco use by current or prior dependence.

### 2.3 Ten-year ASCVD risk score

Ten-year ASCVD risk was estimated using the American College of Cardiology/American Heart Association Pooled Cohort Equations (PCE) as implemented in the RiskScorescvd R package version 0.3.1 (Perez-Vicencio et al., 2025). Required inputs included age, sex, race and ethnicity, total cholesterol, HDL cholesterol, systolic blood pressure, hypertension treatment status, diabetes status, and smoking/tobacco status. Race and ethnicity was coded according to the PCE specification, with NHB participants mapped to the Black risk equation and NHW and Hispanic participants mapped to the “Other” equation, consistent with standard practice when Hispanic-specific coefficients are unavailable. Total cholesterol and HDL cholesterol were converted from mg/dL to mmol/L using the standard conversion factor of 0.02586, as required by the PCE algorithm. Systolic blood pressure was calculated as the mean of two objective systolic blood pressure measurements when both were available; otherwise, a single available measurement was used. Ten-year ASCVD risk scores were calculated in the complete-case analytic cohort prior to modeling using the RiskScorescvd package (Perez-Vicencio et al., 2025). Continuous ASCVD scores were used in analyses. Because race and ethnicity are incorporated directly into the Pooled Cohort Equations, the racial and ethnic diversity of HABS-HD is relevant not only to the generalizability of the study but also to the calculation and interpretation of the ASCVD risk score itself.

### 2.4 Neuroimaging measures

PET scans: Amyloid burden (A) was quantified using [18F] florbetaben PET acquired according to the Alzheimer’s Disease Neuroimaging Initiative florbetaben protocol (Weiner et al., 2017). Global amyloid burden was expressed as a SUVR derived from FreeSurfer-defined cortical regions corresponding to the florbetaben meta-region of interest, including frontal, anterior and posterior cingulate, lateral parietal, and lateral temporal cortex, normalized to whole cerebellum. Increasing SUVR values reflect greater amyloid deposition. Participants were classified according to amyloid PET status using a global florbetaben PET SUVR threshold of 1.08 (Royse et al., 2021). Participants with global amyloid PET SUVR values ≥1.08 were classified as amyloid positive, whereas participants with values <1.08 SUVR were classified as amyloid negative.

Tau pathology (T) was assessed using [18F] PI-2620 PET. Medial temporal lobe (MTL) SUVR values were computed using regions defined by Mormino et al. (Mormino et al., 2021), including the entorhinal cortex, hippocampus, and amygdala, and normalized to an inferior cerebellar gray matter reference region. Increasing SUVR values reflect greater tau accumulation.

MRI: Neuroimaging acquisition, processing, and data-management methods for HABS-HD have been detailed previously (Petersen et al., 2025). All HABS-HD neuroimaging data are centrally stored, managed, and processed through the University of Southern California Laboratory of Neuro Imaging (LONI). MRI data were acquired on 3 T Siemens scanners, including a Magnetom Skyra from March 2017 through December 2020 and a Magnetom Vida from December 2020 onward. The Vida imaging protocol was subsequently modified in August 2024. To account for differences in acquisition across scanner and protocol periods, HABS-HD data releases include indicators for scanner type and acquisition protocol, and MRI scanner/protocol indicators were included as covariates in relevant analyses.

The current study used T1-weighted magnetization-prepared rapid gradient echo (MPRAGE) and fluid-attenuated inversion recovery (FLAIR) MRI sequences to derive cortical thickness, hippocampal volume, and white matter hyperintensity (WMH) volume. On the Skyra protocol, MPRAGE was acquired with TR = 2,300 ms, TE = 2.93 ms, voxel size = 1.1 × 1.1 × 1.2 mm, flip angle = 9°, GRAPPA acceleration = 2, sagittal orientation, and TI = 900 ms; FLAIR was acquired with TR = 4,800 ms, TE = 441 ms, voxel size = 1.0 × 1.0 × 1.2 mm, variable flip angle, GRAPPA acceleration = 3, sagittal orientation, and TI = 1,650 ms. On the Vida protocol used from December 2020 through August 2024, MPRAGE was acquired with TR = 2,300 ms, TE = 2.98 ms, voxel size = 1.0 × 1.0 × 1.0 mm, flip angle = 9°, GRAPPA acceleration = 2, sagittal orientation, and TI = 900 ms; FLAIR was acquired with TR = 4,800 ms, TE = 441 ms, voxel size = 1.0 × 1.0 × 1.2 mm, variable flip angle, GRAPPA acceleration = 3, sagittal orientation, and TI = 1,650 ms. In the modified Vida protocol used beginning August 2024, MPRAGE parameters remained TR = 2,300 ms, TE = 2.98 ms, voxel size = 1.0 × 1.0 × 1.0 mm, flip angle = 9°, GRAPPA acceleration = 2, sagittal orientation, and TI = 900 ms; FLAIR was acquired with TR = 5,000 ms, TE = 393 ms, voxel size = 1.0 × 1.0 × 1.0 mm, variable flip angle, GRAPPA acceleration = 2, sagittal orientation, and TI = 1,700 ms.

Neurodegeneration (N) was indexed using both cortical thickness and hippocampal volume derived from T1-weighted structural MRI. Cortical thickness was estimated using FreeSurfer version 5.3 and summarized as a meta-region of interest computed as a surface area-weighted average of bilateral entorhinal, fusiform, inferior temporal, and middle temporal cortices (Fischl, 2012). Lower cortical thickness values reflect greater neurodegeneration. Hippocampal volume was quantified using HippoDeep, an automated deep learning-based segmentation pipeline (Thyreau et al., 2018). Left and right hippocampal volumes were summed to derive total hippocampal volume. The primary hippocampal outcome was an intracranial volume (ICV)-adjusted hippocampal volume z-score provided through the HABS-HD/LONI processing pipeline. The exact reference distribution and mathematical procedure used to generate this derived *z*-score were not available in the published HABS-HD documentation and are therefore acknowledged as a limitation.

Vascular injury (V) was assessed using WMH volume derived from FLAIR MRI. WMH volume has been examined in prior HABS-HD studies as a marker of vascular brain injury in relation to cardiometabolic risk, blood pressure, sex differences, race and ethnicity, and cognition (Hayes et al., 2025d, 2025c, 2025b, 2026b). WMH segmentation was performed using the Lesion Growth Algorithm implemented in the Lesion Segmentation Toolbox for SPM (Schmidt et al., 2012). Total WMH volume was calculated in milliliters and underwent a quality-control workflow to assess completeness, identify zero-valued observations, evaluate transformed distributions, and detect invalid values. A total of 69 participants had WMH values recorded as 0.000. Because these values could not be directly log-transformed without introducing an arbitrary constant and produced a distinct floor in the transformed distribution, these observations were set to missing for analyses of continuous WMH burden. Among participants with nonzero WMH values, WMH volume was divided by ICV and subsequently log-transformed. The resulting log-transformed ICV-adjusted WMH volume was used as the primary vascular injury outcome.

### 2.5 Cognitive measures and diagnosis

Each individual neuropsychological test score was first standardized within the analytic sample after application of the study inclusion criteria. For each test, a *z*-score was calculated by subtracting the analytic-sample mean from the participant’s raw score and dividing the result by the analytic-sample standard deviation. The resulting test-specific z-scores were then averaged within each prespecified cognitive domain to create a composite domain score, with higher values indicating better cognitive performance. The episodic memory composite was calculated by averaging the standardized scores for the Spanish–English Verbal Learning Test immediate recall, defined as the total score across trials 1–5, Spanish–English Verbal Learning Test delayed recall (González et al., 2002), Logical Memory I, and Logical Memory II (Reitan et al., 1985). The executive function composite was calculated by averaging the standardized Digit Span and Digit Symbol Substitution scores (Lezak et al., 2004). The processing speed composite was calculated by averaging the standardized completion-time scores for Trail Making Test Parts A and B (Lezak et al., 2004). Because longer completion times indicate poorer performance, Trail Making Test scores were multiplied by −1 before standardization so that higher z-scores consistently reflected better performance. The language composite was calculated by averaging the standardized Animal Naming and F-A-S phonemic fluency scores (O’Bryant et al., 2018). Thus, each domain score represented the mean of the standardized scores from all neuropsychological tests assigned to that domain rather than a standardized transformation of an already combined raw score. Identical methodology has been used for HABS-HD previously (Hayes et al., 2025a, 2026a).

### 2.6 Clinical cognitive status

Cognitive status was determined through a standardized multidisciplinary consensus adjudication process based on neuropsychological performance and functional assessment. Participants were classified as cognitively unimpaired or as having mild cognitive impairment (MCI), and individuals with dementia were excluded from analyses (Petersen et al., 2025).

### 2.7 Statistical analysis

Participant characteristics were summarized using descriptive statistics, with continuous variables reported as mean ± standard deviation and categorical variables reported as counts and percentages.

The ten-year ASCVD risk score was analyzed as a continuous predictor in all primary models. Age, sex, and race and ethnicity were not entered as separate covariates because they are components of the PCE and contribute directly to the calculation of the exposure. Additional adjustment for these variables would change the estimate from the association of the clinically used composite ASCVD score to the association of the score conditional on selected components and could introduce statistical redundancy or instability. Accordingly, the reported estimates represent associations with the overall cardiovascular risk profile captured by the pooled cohort equation rather than associations with vascular burden independent of age, sex, or race and ethnicity.

Separate multivariable linear regression models were used to examine the association between continuous ten-year ASCVD risk score and each AT(N)-V neuroimaging biomarker. Global amyloid burden, measured using global florbetaben PET SUVR, was modeled as the outcome with ten-year ASCVD risk score as the primary predictor, with adjustment for *APOE* ε4 carrier status and amyloid PET scanner. MTL tau PET SUVR was modeled as the outcome with ten-year ASCVD risk score as the primary predictor, with adjustment for *APOE* ε4 carrier status. Cortical thickness, ICV-adjusted hippocampal volume z-score, and log-transformed ICV-adjusted WMH volume were modeled as separate outcomes with ten-year ASCVD risk score as the primary predictor, with adjustment for *APOE* ε4 carrier status and MRI scanner/protocol. Thus, five separate neuroimaging models were estimated: (1) global amyloid PET SUVR, (2) MTL tau PET SUVR, (3) cortical thickness, (4) ICV-adjusted hippocampal volume z-score, and (5) log-transformed ICV-adjusted WMH volume. Regression coefficients were reported with 95% confidence intervals (CI) and *p*-values. To account for multiple comparisons across the set of neuroimaging models, *p*-values were adjusted using the Holm family-wise error correction method. Statistical significance was determined using two-sided Holm-adjusted *p*-values <0.05.

Separate multivariable linear regression models were used to examine associations of continuous ten-year ASCVD risk score and neuroimaging biomarkers with domain-specific cognitive performance. Memory, executive function, processing speed, and language composite scores were modeled as separate continuous outcomes. Increasing cognitive domain scores indicated better performance. Each model included continuous ten-year ASCVD risk score, *APOE* ε4 carrier status, global amyloid florbetaben PET SUVR, cortical thickness, ICV-adjusted hippocampal volume z-score, log-transformed ICV-adjusted WMH volume, amyloid PET scanner, and MRI scanner/protocol. MTL tau PET SUVR was not included in the primary cognitive models because tau PET was available only for a subset of participants, and inclusion of MTL tau would have substantially reduced the complete-case analytic sample. Therefore, the primary cognitive models estimated associations between ten-year ASCVD risk and domain-specific cognitive performance after accounting for amyloid burden, neurodegeneration, cerebrovascular injury, *APOE* ε4 carrier status, and imaging scanner/protocol covariates. Regression coefficients were reported with 95% CI and Holm-adjusted *p*-values.

To provide additional context for interpreting tau-related findings, analyses were repeated after stratifying participants by amyloid PET status. Participants were classified as amyloid positive if global amyloid PET SUVR was ≥1.08 and amyloid negative if global amyloid PET SUVR was <1.08. Within amyloid-positive and amyloid-negative groups, separate multivariable linear regression models were used to examine the association between continuous ten-year ASCVD risk score and each AT(N)-V neuroimaging biomarker. The global amyloid PET SUVR model included continuous ten-year ASCVD risk score, *APOE* ε4 carrier status, and amyloid PET scanner. The MTL tau PET SUVR model included continuous ten-year ASCVD risk score and *APOE* ε4 carrier status. The cortical thickness, ICV-adjusted hippocampal volume z-score, and log-transformed ICV-adjusted WMH volume models each included continuous ten-year ASCVD risk score, *APOE* ε4 carrier status, and MRI scanner/protocol. The *p*-values were adjusted using the Holm family-wise error correction method within each amyloid-status group.

Finally, amyloid-stratified cognitive models were fitted to examine associations of ten-year ASCVD risk score and neuroimaging biomarkers with domain-specific cognitive performance separately among amyloid-positive and amyloid-negative participants. These models used the same covariate structure as the primary cognitive models and included continuous ten-year ASCVD risk score, *APOE* ε4 carrier status, global amyloid PET SUVR, cortical thickness, ICV-adjusted hippocampal volume z-score, log-transformed ICV-adjusted WMH volume, amyloid PET scanner, and MRI scanner/protocol. Regression coefficients were reported with 95% CI and Holm-adjusted *p*-values.

All analyses were conducted in R version 4.2.3.

Data Availability Statement: HABS-HD data are available upon request through the Laboratory of Neuro Imaging (LONI) data portal^1^.

## 3 Results

### 3.1 Participant characteristics

Participant characteristics for the overall analytic sample are presented in Table 1. The analytic sample included 1,626 participants from HABS-HD with available baseline clinical assessment, ten-year ASCVD risk score inputs, and amyloid PET imaging corresponding to Visit 1. The mean ten-year ASCVD risk score was 0.15 ± 0.13, with a median score of 0.11. The mean age of the sample was 63.5 ± 8.16 years, and 569 participants were male (35.0%). The sample was racially and ethnically diverse, including 810 NHB participants (49.8%), 424 Hispanic participants (26.1%), and 392 NHW participants (24.1%).

Cardiometabolic risk factors were common in the analytic sample. Diabetes was present in 401 participants (24.7%), hypertension in 1,095 participants (67.3%), and tobacco dependence in 136 participants (8.4%). *APOE* ε4 carrier status was present in 519 participants (31.9%), with missing *APOE* ε4 data for 87 participants (5.4%). Mean global amyloid PET SUVR was 1.03 ± 0.13, and 247 participants (15.2%) were classified as amyloid positive using the global amyloid PET SUVR threshold of ≥1.08. Mean MTL tau SUVR was 1.13 ± 0.12 among participants with available tau PET data; 749 participants (46.1%) were missing MTL tau SUVR. The mean cortical thickness was 2.78 ± 0.12, mean hippocampal volume was 6.45 ± 0.75 mL, mean ICV-adjusted hippocampal volume *z*-score was 0.07 ± 0.94, and mean log-transformed ICV-adjusted WMH volume was −7.38 ± 1.60. MCI was present in 435 participants (26.8%).

### 3.2 Associations between ten-year ASCVD risk and AT(N)V neuroimaging biomarkers

Increasing ten-year ASCVD risk was associated with neuroimaging markers across the AT(N)V framework (Figure 1). Increasing ten-year ASCVD risk was positively associated with global amyloid PET SUVR (*β* = 0.19, 95% CI [0.14 to 0.24], Holm-adjusted *p* < 0.0001) and MTL tau SUVR (*β* = 0.14, 95% CI [0.07 to 0.20], Holm-adjusted *p* < 0.0001). Increasing ten-year ASCVD risk was also associated with smaller cortical thickness (*β* = −0.35, 95% CI [−0.40 to −0.31], Holm-adjusted *p* < 0.0001) and smaller ICV-adjusted hippocampal volume (*β* = −2.91, 95% CI [−3.26 to −2.56], Holm-adjusted *p* < 0.0001). In addition, increasing ten-year ASCVD risk was associated with greater vascular injury, reflected by increasing log-transformed ICV-adjusted WMH volume (*β* = 5.11, 95% CI [4.52 to 5.69], Holm-adjusted *p* < 0.0001).

**Figure 1:**
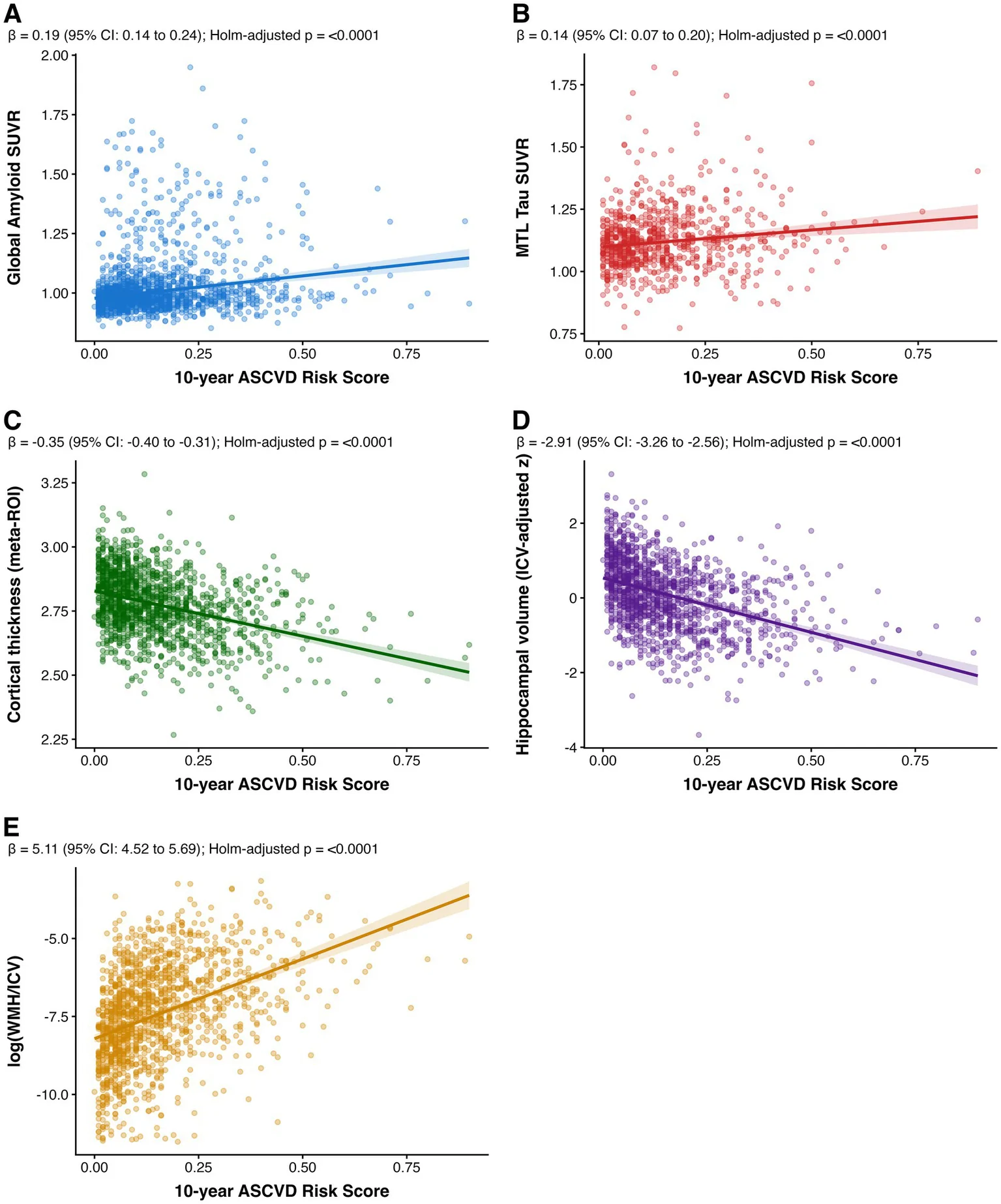
Associations of ten-year ASCVD risk score with amyloid, tau, neurodegeneration, and cerebrovascular imaging markers. Scatter plots with adjusted regression lines showing associations between ASCVD risk score and neuroimaging outcomes. **A** (global amyloid PET SUVR) was modeled as a function of ASCVD risk score, amyloid PET scanner, and APOE ε4 status. **B** (MTL tau PET SUVR) was modeled as a function of ASCVD risk score and APOE ε4 status. **C–E** (cortical thickness, ICV-adjusted hippocampal volume *z*-score, and log-transformed ICV-adjusted WMH volume) were each modeled as a function of ASCVD risk score, MRI scanner/protocol, and APOE ε4 status.

### 3.3 Associations between ten-year ASCVD risk, neuroimaging biomarkers, and cognitive domains

Associations of ten-year ASCVD risk, *APOE* ε4 status, AT(N)V neuroimaging biomarkers, and scanner covariates with domain-specific cognitive performance are presented in Table 2. In multivariable models, increasing ten-year ASCVD risk was consistently associated with poorer cognitive performance across all domains after accounting for *APOE* ε4 status, global amyloid PET SUVR, cortical thickness, ICV-adjusted hippocampal volume, ICV-adjusted WMH volume, PET scanner, and MRI scanner. Specifically, increasing ten-year ASCVD risk was associated with reduced memory performance (*β* = −1.08, 95% CI [−1.51 to −0.64], Holm-adjusted *p* < 0.001), executive function (*β* = −1.32, 95% CI [−1.74 to −0.91], Holm-adjusted *p* < 0.001), processing speed (*β* = −1.28, 95% CI [−1.74 to −0.82], Holm-adjusted *p* < 0.001), and language performance (*β* = −0.92, 95% CI [−1.35 to −0.49], Holm-adjusted *p* < 0.001).

**Table 2:** Associations of ten-year atherosclerotic cardiovascular disease risk score and AT(N)-V neuroimaging markers with domain-specific cognitive performance.

Predictors	Memory		Executive function		Processing speed		Language	
	*β* (95% CI)	*p*	*β* (95% CI)	*p*	*β* (95% CI)	*p*	*β* (95% CI)	*p*
ASCVD score	−1.08 (−1.51–−0.64)	**<0.001**	−1.32 (−1.74–−0.91)	**<0.001**	−1.28 (−1.74–−0.82)	**<0.001**	−0.92 (−1.35–−0.49)	**<0.001**
APOE ε4	−0.01 (−0.12–0.09)	1.000	0.07 (−0.03–0.17)	0.761	0.08 (−0.02–0.19)	0.638	0.07 (−0.04–0.17)	1.000
Global Amyloid SUVR	−0.02 (−0.39–0.35)	1.000	−0.18 (−0.53–0.18)	0.831	−0.48 (−0.86–−0.09)	0.108	0.15 (−0.22–0.51)	1.000
Cortical Thickness	−0.01 (−1.23–1.21)	1.000	−1.26 (−2.43–−0.09)	0.280	−0.82 (−2.10–0.46)	0.680	−0.12 (−1.34–1.10)	1.000
Normalized HCV (z)	0.34 (−0.10–0.78)	1.000	0.34 (−0.07–0.76)	0.639	0.75 (0.29–1.20)	**0.013**	0.09 (−0.35–0.53)	1.000
Amyloid PET Scanner [MCT20]	0.15 (0.09–0.21)	**<0.001**	0.03 (−0.02–0.09)	0.831	0.06 (−0.00–0.12)	0.417	0.05 (−0.00–0.11)	0.592
Normalized WMH Volume	0.01 (−0.03–0.04)	1.000	−0.04 (−0.07–−0.00)	0.239	−0.05 (−0.09–−0.02)	**0.042**	−0.03 (−0.06–0.01)	0.911
MRI Scanner [Vida1]	0.17 (−1.01–1.34)	1.000	−0.82 (−1.95–0.30)	0.761	−0.54 (−1.77–0.69)	0.680	0.15 (−1.02–1.32)	1.000
MRI Scanner [Vida1a]	0.25 (−1.05–1.55)	1.000	−1.22 (−2.46–0.02)	0.380	−0.87 (−2.22–0.49)	0.680	−0.12 (−1.42–1.17)	1.000
Observations	1,218		1,218		1,217		1,218	
*R*^2^/*R*^2^ adjusted	0.082/0.075		0.095/0.088		0.116/0.109		0.047/0.040	

Separate multivariable linear regression models were fitted for memory, executive function, processing speed, and language composite scores. Each model simultaneously included the continuous 10-year atherosclerotic cardiovascular disease (ASCVD) risk score, APOE ε4 carrier status, global amyloid positron emission tomography standardized uptake value ratio (SUVR), cortical thickness, normalized hippocampal volume z-score, normalized white matter hyperintensity (WMH) volume, PET scanner, and MRI scanner. Regression coefficients are reported as β estimates with 95% confidence intervals and Holm adjusted p-values. For continuous predictors, *β* represents the difference in cognitive-domain score associated with a one-unit increase in the predictor; for categorical predictors, *β* represents the difference relative to the reference category. Higher cognitive scores indicate better performance. Scanner coefficients are relative to the omitted reference scanner category. Model-specific sample sizes and unadjusted and adjusted *R*^2^ values are reported. APOE, apolipoprotein E; ASCVD, atherosclerotic cardiovascular disease; PET, positron emission tomography; SUVR, standardized uptake value ratio; WMH, white matter hyperintensity.

Among the neuroimaging biomarkers included in these models, increased ICV-adjusted hippocampal volume was associated with better processing speed (*β* = 0.75, 95% CI [0.29 to 1.20], Holm-adjusted *p* = 0.013), whereas increased ICV-adjusted WMH volume was associated with poorer processing speed (*β* = −0.05, 95% CI [−0.09 to −0.02], Holm-adjusted *p* = 0.042). Global amyloid PET SUVR, cortical thickness, and *APOE* ε4 status were not significantly associated with cognitive performance after Holm adjustment in the overall analytic sample.

### 3.4 Amyloid-stratified associations between ten-year ASCVD risk and AT(N)V neuroimaging biomarkers

Amyloid-stratified analyses were conducted to examine associations between ten-year ASCVD risk and AT(N)V neuroimaging biomarkers within amyloid PET status strata (Figure 2). Among amyloid-positive participants, increasing ten-year ASCVD risk was not significantly associated with global amyloid PET SUVR (*n* = 235; *β* = 0.11, 95% CI [−0.05 to 0.27], Holm-adjusted *p* = 0.3692) or MTL tau PET SUVR (*n* = 129; *β* = 0.15, 95% CI [−0.09 to 0.38], Holm-adjusted *p* = 0.3692). However, increasing 10-year ASCVD risk was significantly associated with reduced cortical thickness (*n* = 222; *β* = −0.27, 95% CI [−0.38 to −0.16], Holm-adjusted *p* < 0.0001), reduced ICV-adjusted hippocampal volume *z*-score (*n* = 209; *β* = −2.33, 95% CI [−3.16 to −1.50], Holm-adjusted *p* < 0.0001), and increased log-transformed ICV-adjusted WMH volume (*n* = 223; *β* = 3.68, 95% CI [2.31 to 5.05], Holm-adjusted *p* < 0.0001).

**Figure 2:**
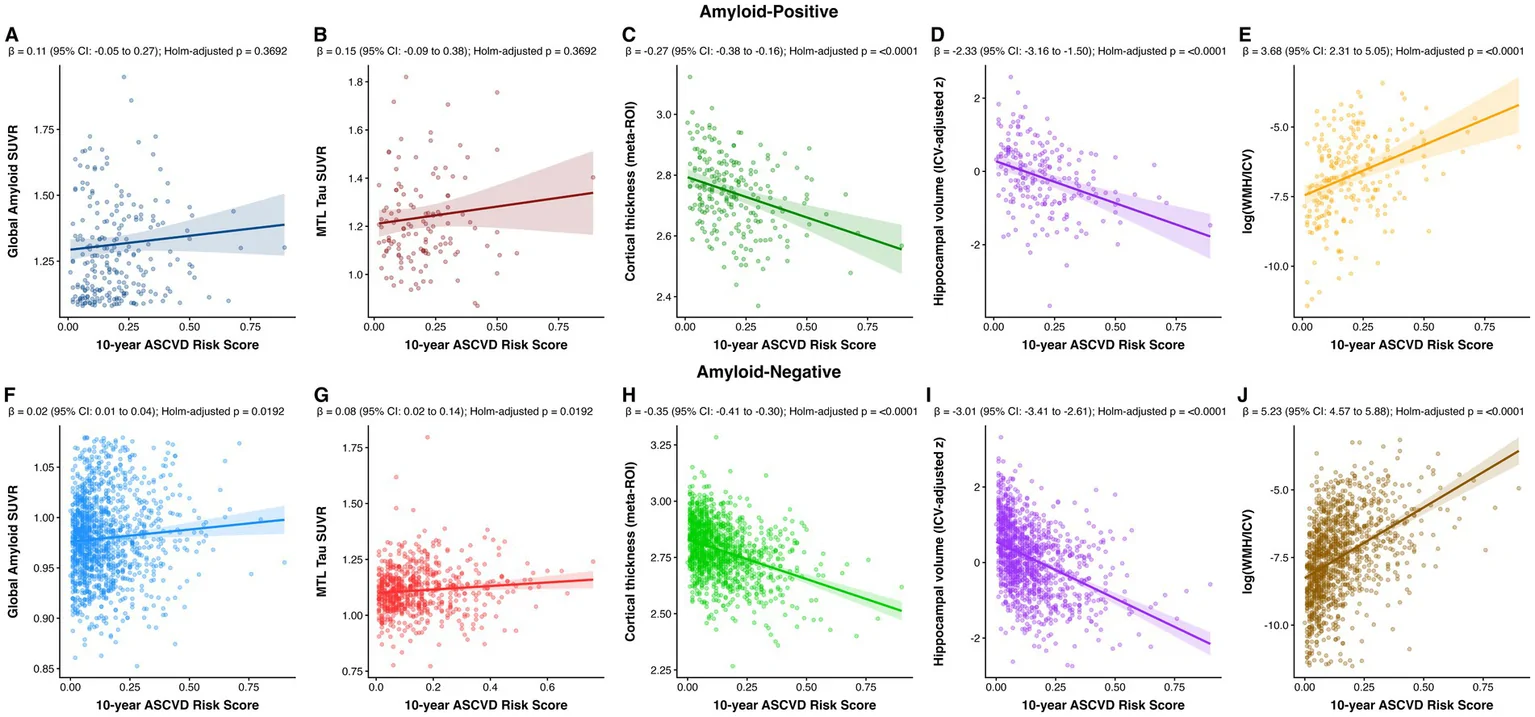
Associations of ten-year ASCVD risk score with amyloid, tau, neurodegeneration, and cerebrovascular imaging markers stratified by global amyloid positivity. **A** and **F** (global amyloid PET SUVR) were modeled as a function of ASCVD risk score, amyloid PET scanner, and APOE ε4 status. **B** and **G** (MTL tau PET SUVR) were modeled as a function of ASCVD risk score and APOE ε4 status. **C–E** and **H–J** (cortical thickness, ICV-adjusted hippocampal volume z-score, and log-transformed ICV-adjusted WMH volume) were each modeled as a function of ASCVD risk score, MRI scanner/protocol, and APOE ε4 status.

Among amyloid-negative participants, increasing ten-year ASCVD risk was significantly associated with increasing global amyloid PET SUVR (*n* = 1,303; *β* = 0.02, 95% CI [0.01 to 0.04], Holm-adjusted *p* = 0.0192) and MTL tau PET SUVR (*n* = 694; *β* = 0.08, 95% CI [0.02 to 0.14], Holm-adjusted *p* = 0.0192), although these associations were modest in magnitude. Increasing ten-year ASCVD risk was also associated with smaller cortical thickness (*n* = 1,214; *β* = −0.35, 95% CI [−0.41 to −0.30], Holm-adjusted *p* < 0.0001), reduced ICV-adjusted hippocampal volume z-score (*n* = 1,151; *β* = −3.01, 95% CI [−3.41 to −2.61], Holm-adjusted *p* < 0.0001), and increased log-transformed ICV-adjusted WMH volume (*n* = 1,242; *β* = 5.23, 95% CI [4.57 to 5.88], Holm-adjusted *p* < 0.0001).

Overall, amyloid-stratified analyses showed that associations between increasing ten-year ASCVD risk and structural and vascular injury markers were evident in both amyloid-positive and amyloid-negative participants. In contrast, associations with amyloid and MTL tau PET were modest and statistically significant only among amyloid-negative participants. These stratified analyses provide additional context for interpreting MTL tau findings, given that medial temporal tau signal may reflect both AD-related and non-AD age-related tau processes, particularly among amyloid-negative participants.

### 3.5 Amyloid-positive associations between ten-year ASCVD risk, AT(N)V biomarkers, and cognitive domains

Associations between ten-year ASCVD risk, AT(N)V neuroimaging biomarkers, and domain-specific cognitive performance among amyloid-positive participants are presented in Table 3. In amyloid-positive participants, increasing ten-year ASCVD risk was associated with reduced executive function (*β* = −1.80, 95% CI [−2.71 to −0.90], *p* = 0.001) and reduced processing speed scores (*β* = −1.82, 95% CI [−3.07 to −0.56], *p* = 0.047) after Holm adjustment. Associations between ten-year ASCVD risk and memory (*β* = −1.04, 95% CI [−2.09 to 0.01], *p* = 0.465) and language performance (*β* = −1.37, 95% CI [−2.37 to −0.37], *p* = 0.075) did not remain statistically significant after correction. ICV-adjusted hippocampal volume was positively associated with memory performance among amyloid-positive participants (*β* = 0.31, 95% CI [0.14 to 0.47], *p* = 0.002). Other AT(N)V biomarkers were not significantly associated with cognitive performance after Holm adjustment in the amyloid-positive subgroup.

**Table 3:** Associations of ten-year atherosclerotic cardiovascular disease risk score and AT(N)-V neuroimaging markers with domain-specific cognitive performance in amyloid positive participants.

Predictors	Memory		Executive Function		Processing Speed		Language	
	*β* (95% CI)	*p*	*β* (95% CI)	*p*	*β* (95% CI)	*p*	*β* (95% CI)	*p*
ASCVD score	−1.04 (−2.09–0.01)	0.465	−1.80 (−2.71–−0.90)	**0.001**	−1.82 (−3.07–−0.56)	**0.047**	−1.37 (−2.37–−0.37)	0.075
APOE ε4	−0.13 (−0.40–0.15)	1.000	0.07 (−0.17–0.31)	1.000	0.09 (−0.24–0.42)	1.000	0.00 (−0.26–0.27)	1.000
Global Amyloid SUVR	0.02 (−0.77–0.80)	1.000	−0.18 (−0.86–0.49)	1.000	−0.06 (−1.00–0.87)	1.000	0.52 (−0.22–1.27)	1.000
Amyloid PET Scanner [MCT20]	−0.18 (−2.34–1.98)	1.000	−1.03 (−2.90–0.84)	1.000	−0.26 (−2.85–2.33)	1.000	−0.26 (−2.33–1.80)	1.000
Cortical Thickness	0.09 (−1.12–1.30)	1.000	0.09 (−0.96–1.13)	1.000	0.25 (−1.21–1.70)	1.000	0.05 (−1.11–1.20)	1.000
Normalized HCV (z)	0.31 (0.14–0.47)	**0.002**	0.16 (0.02–0.30)	0.214	0.19 (−0.00–0.38)	0.453	0.18 (0.02–0.33)	0.209
Normalized WMH Volume	−0.03 (−0.13–0.07)	1.000	0.05 (−0.03–0.14)	1.000	−0.04 (−0.16–0.08)	1.000	−0.07 (−0.17–0.02)	1.000
MRI Scanner [Vida1]	0.19 (−1.69–2.06)	1.000	−0.80 (−2.42–0.82)	1.000	−0.67 (−2.92–1.57)	1.000	−0.10 (−1.89–1.69)	1.000
MRI Scanner [Vida1a]	0.90 (−1.26–3.06)	1.000	−0.79 (−2.66–1.07)	1.000	−0.35 (−2.93–2.23)	1.000	−0.74 (−2.80–1.32)	1.000
Observations	197		197		197		197	
*R*^2^/*R*^2^ adjusted	0.161/0.121		0.158/0.118		0.127/0.084		0.150/0.109	

Separate multivariable linear regression models were fitted for memory, executive function, processing speed, and language composite scores in participants with an amyloid-positive PET scan based on a global amyloid PET SUVR ≥1.08. Each model simultaneously included the continuous ten-year atherosclerotic cardiovascular disease (ASCVD) risk score, APOE ε4 carrier status, global amyloid positron emission tomography standardized uptake value ratio (SUVR), cortical thickness, normalized hippocampal volume z-score, normalized white matter hyperintensity (WMH) volume, PET scanner, and MRI scanner. Regression coefficients are reported as *β* estimates with 95% confidence intervals and Holm adjusted *p*-values. For continuous predictors, *β* represents the difference in cognitive-domain score associated with a one-unit increase in the predictor; for categorical predictors, *β* represents the difference relative to the reference category. Higher cognitive scores indicate better performance. Scanner coefficients are relative to the omitted reference scanner category. Model-specific sample sizes and unadjusted and adjusted *R*^2^ values are reported. APOE, apolipoprotein E; ASCVD, atherosclerotic cardiovascular disease; PET, positron emission tomography; SUVR, standardized uptake value ratio; WMH, white matter hyperintensity.

### 3.6 Amyloid-negative associations between ten-year ASCVD risk, AT(N)V biomarkers, and cognitive domains

Associations between ten-year ASCVD risk, AT(N)V neuroimaging biomarkers, and domain-specific cognitive performance among amyloid-negative participants are presented in Table 4. In amyloid-negative participants, increasing ten-year ASCVD risk was associated with decreased performance across all cognitive domains, including memory (*β* = −1.11, 95% CI [−1.60 to −0.63], *p* < 0.001), executive function (*β* = −1.18, 95% CI [−1.65 to −0.71], *p* < 0.001), processing speed (*β* = −1.10, 95% CI [−1.59 to −0.61], *p* < 0.001), and language (*β* = −0.76, 95% CI [−1.25 to −0.28], *p* = 0.021).

**Table 4:** Associations of ten-year atherosclerotic cardiovascular disease risk score and AT(N)-V neuroimaging markers with domain-specific cognitive performance in amyloid negative participants.

Predictors	Memory		Executive function		Processing speed		Language	
	*β* (95% CI)	*p*	*β* (95% CI)	*p*	*β* (95% CI)	*p*	*β* (95% CI)	*p*
ASCVD score	−1.11 (−1.60–−0.63)	**<0.001**	−1.18 (−1.65–−0.71)	**<0.001**	−1.10 (−1.59–−0.61)	**<0.001**	−0.76 (−1.25–−0.28)	**0.021**
APOE ε4	0.01 (−0.10–0.13)	1.000	0.07 (−0.04–0.18)	1.000	0.09 (−0.03–0.20)	0.797	0.07 (−0.04–0.19)	1.000
Global Amyloid SUVR	1.37 (0.11–2.62)	0.262	−0.12 (−1.35–1.11)	1.000	−0.19 (−1.45–1.07)	1.000	−0.37 (−1.63–0.89)	1.000
Amyloid PET Scanner [MCT20]	0.01 (−1.64–1.67)	1.000	−1.34 (−2.96–0.28)	0.702	−0.86 (−2.52–0.81)	1.000	0.01 (−1.65–1.68)	1.000
Cortical Thickness	0.38 (−0.09–0.85)	0.668	0.42 (−0.04–0.88)	0.576	0.87 (0.40–1.34)	**0.003**	0.10 (−0.38–0.57)	1.000
Normalized HCV (z)	0.12 (0.06–0.18)	**0.002**	0.01 (−0.06–0.07)	1.000	0.03 (−0.03–0.10)	1.000	0.03 (−0.03–0.10)	1.000
Normalized WMH Volume	0.01 (−0.03–0.05)	1.000	−0.06 (−0.09–−0.02)	**0.022**	−0.06 (−0.09–−0.02)	**0.028**	−0.02 (−0.06–0.01)	1.000
MRI Scanner [Vida1]	0.15 (−1.47–1.77)	1.000	−0.87 (−2.46–0.72)	1.000	−0.48 (−2.11–1.15)	1.000	0.29 (−1.34–1.92)	1.000
MRI Scanner [Vida1a]	−0.09 (−1.84–1.66)	1.000	−1.44 (−3.15–0.28)	0.702	−1.11 (−2.87–0.65)	1.000	0.21 (−1.55–1.97)	1.000
Observations	1,021		1,021		1,020		1,021	
*R*^2^/*R*^2^ adjusted	0.074/0.065		0.087/0.079		0.105/0.097		0.033/0.025	

Separate multivariable linear regression models were fitted for memory, executive function, processing speed, and language composite scores in participants with an amyloid-negative PET scan based on a global amyloid PET SUVR <1.08. Each model simultaneously included the continuous 10-year atherosclerotic cardiovascular disease (ASCVD) risk score, APOE ε4 carrier status, global amyloid positron emission tomography standardized uptake value ratio (SUVR), cortical thickness, normalized hippocampal volume z-score, normalized white matter hyperintensity (WMH) volume, PET scanner, and MRI scanner. Regression coefficients are reported as *β* estimates with 95% confidence intervals and Holm adjusted *p*-values. For continuous predictors, *β* represents the difference in cognitive-domain score associated with a one-unit increase in the predictor; for categorical predictors, *β* represents the difference relative to the reference category. Higher cognitive scores indicate better performance. Scanner coefficients are relative to the omitted reference scanner category. Model-specific sample sizes and unadjusted and adjusted *R*^2^ values are reported. APOE, apolipoprotein E; ASCVD, atherosclerotic cardiovascular disease; PET, positron emission tomography; SUVR, standardized uptake value ratio; WMH, white matter hyperintensity.

Among neuroimaging biomarkers, cortical thickness was positively associated with processing speed (β = 0.87, 95% CI [0.40 to 1.34], *p* = 0.003), and ICV-adjusted hippocampal volume was positively associated with memory performance (*β* = 0.12, 95% CI [0.06 to 0.18], *p* = 0.002). ICV-adjusted WMH volume was negatively associated with executive function (*β* = −0.06, 95% CI [−0.09 to −0.02], *p* = 0.022) and processing speed (*β* = −0.06, 95% CI [−0.09 to −0.02], *p* = 0.028). *APOE* ε4 status and global amyloid PET SUVR were not significantly associated with cognitive performance after Holm adjustment in the amyloid-negative subgroup.

## 4 Discussion

In this cross-sectional study of a large, racially and ethnically diverse cohort, increases in ten-year ASCVD risk was associated with greater AT(N)V biomarker burden, including increased global amyloid SUVR, MTL tau SUVR, reduced cortical thickness, smaller hippocampal volume, and increased WMH volume. Increases in ten-year ASCVD risk was also consistently associated with poorer performance across multiple cognitive domains after accounting for *APOE* ε4 status, amyloid burden, neurodegeneration, cerebrovascular injury, and imaging scanner covariates. These findings suggest that ten-year ASCVD risk may capture clinically relevant variation in cardiovascular and brain health burden that is not fully reflected by individual neuroimaging biomarkers alone.

A key finding of this study is that ten-year ASCVD risk demonstrated broad associations across the full AT(N)V spectrum, with the strongest effects observed for WMH volume and structural neurodegeneration. This pattern is consistent with prior work showing that vascular risk is closely tied to cerebrovascular injury and brain atrophy in aging populations. For example, Rabin et al. (Rabin et al., 2022) showed that increased vascular risk was associated with greater brain atrophy over time, while Gottesman et al. (2017b) demonstrated that midlife vascular risk contributes meaningfully to later cognitive decline and dementia risk. Our findings extend this literature by showing that ten-year ASCVD risk, as an integrated measure of cardiovascular burden, is associated not only with vascular injury but also with amyloid and tau pathology. At the same time, the comparatively weaker associations with amyloid and tau suggest that ten-year ASCVD risk may be more strongly associated with cerebrovascular injury and structural brain changes than with AD-related pathology in this cohort. Clinically, these modest amyloid and tau associations should not be interpreted as indicating that ten-year ASCVD risk is a standalone proxy for AD proteinopathy. Rather, the findings suggest that increases in ten-year ASCVD risk may co-occur with slightly elevated amyloid and MTL tau burden at the group level, while the larger coefficients observed with WMH volume and structural measures may be more relevant for cardiovascular risk–based brain health monitoring in this cohort. These findings should be interpreted as associations rather than evidence of a specific biological mechanism. Although prior neurovascular models provide important biological context (Zlokovic, 2011; Iadecola, 2017), the present study did not directly measure vascular dysfunction, blood–brain barrier integrity, amyloid clearance, neuroinflammation, or cerebral hypoperfusion.

Beyond its associations with neuroimaging biomarkers, increases in ten-year ASCVD risk was also associated with poorer domain-specific cognitive performance. These associations remained evident after adjustment for *APOE* ε4 status and selected neuroimaging measures, suggesting that the imaging biomarkers included in the current models did not fully account for the relationship between ten-year ASCVD risk and cognition. However, these findings should not be interpreted as evidence of a specific vascular mechanism or as demonstrating independence from all possible brain health pathways. Rather, ten-year ASCVD risk may capture broader cardiovascular and metabolic risk information that is not completely represented by the available neuroimaging measures. Prior studies have similarly linked vascular risk burden with cognitive outcomes, and vascular cognitive impairment frameworks provide clinical context for interpreting the relevance of cardiovascular health to cognition (Luchsinger et al., 2005; Iadecola et al., 2019; Levine et al., 2023).

In our multivariable cognitive models, global amyloid SUVR was not significantly associated with cognitive performance after Holm adjustment. In contrast, structural and vascular measures showed more domain-specific associations, with increasing ICV-adjusted hippocampal volume being associated with increasing processing speed and increased WMH volume associated with lower processing speed scores. This pattern is broadly consistent with biomarker models in which amyloid burden alone is often less closely aligned with cognitive performance than downstream markers of neurodegeneration or cerebrovascular injury (Jagust, 2018) (Jack et al., 2013; Shirzadi et al., 2024). Ten-year ASCVD risk demonstrated broader associations across cognitive domains, with significant inverse associations with memory, executive function, processing speed, and language. The association of both hippocampal volume and WMH volume with processing speed is also consistent with the sensitivity of processing-speed measures to structural and vascular brain changes in aging (Silbert, 2024). However, the present cross-sectional analyses do not identify the biological mechanisms underlying these associations, and the findings should be interpreted as evidence of association rather than evidence that vascular risk contributes to cognitive decline through pathways distinct from AD biomarkers.

Within this context, the present findings are best interpreted within broader models recognizing that vascular and neurodegenerative processes often co-occur in cognitive aging, rather than as evidence of a single AD-specific pathway. Prior work on vascular cognitive impairment and dementia highlights the relevance of cardiovascular and cerebrovascular health for late-life cognitive outcomes, while recent frameworks emphasize that vascular and neurodegenerative processes may jointly shape clinical expression in aging populations (Iadecola, 2013; Santisteban and Iadecola, 2025). Our findings are consistent with this literature by showing that a clinically used ten-year ASCVD risk score estimate is associated with multiple neuroimaging markers and domain-specific cognitive performance. However, because the current study was cross-sectional and did not directly measure vascular dysfunction, neuroinflammation, amyloid clearance, or intervention response, these findings should be viewed as hypothesis-generating and should not be interpreted as demonstrating that ten-year ASCVD risk causes cognitive decline or operates through a specific biological pathway. Nevertheless, the association of ten-year ASCVD risk with cognition is clinically relevant because cardiovascular risk is potentially modifiable, and multidomain prevention studies support the broader importance of targeting modifiable risk profiles to preserve cognitive health (Kivipelto et al., 2018).

Approximately 46.1% of participants did not have tau PET data. Tau PET imaging is an optional component of HABS-HD rather than a requirement for study participation, and its availability may depend on participant willingness, imaging eligibility, health status, scheduling, and other logistical factors. Therefore, the missing tau PET data cannot be assumed to be missing completely at random, and selection bias may have influenced estimates derived from the tau PET subset. Because the missingness mechanism was not formally evaluated, the tau-related findings should be interpreted as applying to participants with available tau PET imaging and require confirmation in cohorts with more complete ascertainment.

The present findings are also relevant in the context of a racially and ethnically diverse cohort with varying levels of cardiovascular risk. Disparities in cardiovascular disease burden are well documented and may contribute to differences in cognitive aging and dementia risk across populations (Alzheimers Dement, 2026). Although this study was not designed to test racial or ethnic differences directly, the strong associations between ten-year ASCVD risk and multiple domains of brain health underscore the importance of considering vascular burden when studying cognitive aging in diverse populations. More broadly, these findings support efforts to incorporate vascular and metabolic factors into models of AD risk and progression, particularly in historically underrepresented groups (Livingston et al., 2020; Adkins-Jackson et al., 2023; Daniel et al., 2023). Although the racial and ethnic diversity of HABS-HD is an important strength, we did not evaluate race and ethnicity as an effect modifier because the PCE incorporate race-specific coefficients in the calculation of ten-year ASCVD risk score. Consequently, stratified or interaction estimates could reflect differences in the construction, scaling, or calibration of the risk score rather than underlying heterogeneity in the association between cardiovascular risk and brain health outcomes. Future studies should compare findings across multiple cardiovascular risk algorithms, including race- and ethnicity-agnostic equations, to determine whether associations with AT(N)-V biomarkers and cognitive outcomes are consistent across racial and ethnic populations.

The observed associations between ten-year ASCVD risk, neuroimaging biomarkers, and cognitive performance suggest that cardiovascular risk may capture information relevant to multiple dimensions of brain aging. However, the cross-sectional design and available measures do not permit conclusions regarding temporal sequence, causality, or the biological mechanisms underlying these associations. The exclusion of participants with prevalent cardiovascular disease was necessary because the pooled cohort equations are intended for ten-year ASCVD risk estimation in individuals without established clinical cardiovascular disease. This restriction, along with the exclusion of participants with dementia, limits generalizability to the broader aging population, including individuals with existing cardiovascular disease or more advanced cognitive impairment. The reported associations should therefore be interpreted as applying primarily to a primary-prevention population without clinically recognized cardiovascular disease or dementia at baseline, and future studies should evaluate these associations across a wider spectrum of cardiovascular and cognitive health.

The PCE incorporates age, sex, race, cholesterol, blood pressure, antihypertensive treatment, smoking, and diabetes into a single estimate of ten-year cardiovascular risk. Consequently, the observed associations should be interpreted as associations with the composite cardiovascular risk profile represented by the equation, rather than as the isolated association of modifiable vascular burden independent of demographic characteristics. Some associations with neuroimaging and cognitive outcomes may reflect the influence of age and other demographic components embedded within the ten-year ASCVD score. Ten-year ASCVD risk also reflects an estimated 10-year cardiovascular risk rather than direct measures of subclinical atherosclerosis, which may introduce measurement imprecision (Muntner et al., 2014). Future studies should compare multiple cardiovascular risk algorithms, including race-agnostic equations, and evaluate individual or non-demographic cardiovascular risk components to clarify whether associations are primarily attributable to modifiable vascular factors or to the demographic structure of the risk score.

Several additional limitations should be considered. Tau PET imaging was available for only a subset of participants because completion of tau PET is an optional component of HABS-HD and is not required for study participation. Participants who completed tau PET may differ from those without tau PET data because of health status, willingness to undergo imaging, eligibility, scheduling, or other measured and unmeasured factors. Accordingly, selection bias cannot be excluded, and the reduced sample size may have limited the precision and generalizability of associations involving medial temporal lobe tau burden. The tau-related findings should therefore be interpreted as applying to the available imaging subset and require confirmation in studies with more complete tau PET ascertainment. Additionally, hippocampal volume was analyzed using an ICV-adjusted z-score derived from the HABS-HD/LONI processing pipeline. Although this measure provides an internally standardized hippocampal volume outcome, the exact reference distribution and mathematical procedure used to generate the *z*-score were not available. Therefore, hippocampal volume findings should be interpreted with this limitation in mind, and future studies should report the full standardization procedure for derived neuroimaging measures.

Finally, residual confounding from unmeasured or incompletely characterized factors remains possible. Physical activity, dietary patterns, socioeconomic conditions, healthcare access, and detailed medication use, or treatment intensity were not comprehensively incorporated into the current models, although several clinical risk factors and treatment indicators are represented within the ten-year ASCVD risk calculation. Because these factors may influence both cardiovascular risk and brain health, the observed associations may partly reflect broader lifestyle, social, and treatment-related pathways. Longitudinal studies with repeated neuroimaging, cognitive assessments, and more comprehensive measures of modifiable risk factors are needed to determine whether higher ten-year ASCVD risk precedes subsequent changes in brain biomarkers and cognition or instead reflects parallel disease processes.

## 5 Conclusion

In conclusion, higher 10-year ASCVD risk was associated with greater AT(N)-V biomarker burden and poorer domain-specific cognitive performance in this cross-sectional analysis of HABS-HD. These findings identify 10-year ASCVD risk as a potential marker of co-occurring cardiovascular and brain health burden but do not establish causality or temporal direction. The associations observed across AT(N)-V and cognition underscore the importance of considering vascular risk when studying heterogeneity in cognitive aging and dementia-related processes. Longitudinal studies with repeated neuroimaging and cognitive assessments are needed to determine whether higher 10-year ASCVD risk is associated with subsequent progression of amyloid and tau pathology, neurodegeneration, cerebrovascular injury, and cognitive decline.

## Data Availability

The original contributions presented in the study are included in the article/supplementary material, further inquiries can be directed to the corresponding authors.

## Footnotes

1
https://loni.usc.edu/
